# Determinants of HbA1c reduction with FreeStyle Libre flash glucose monitoring (FLARE-NL 5)

**DOI:** 10.1016/j.jcte.2020.100237

**Published:** 2020-10-12

**Authors:** A. Lameijer, M.J. Fokkert, M.A. Edens, R.J. Slingerland, H.J.G. Bilo, P.R. van Dijk

**Affiliations:** a University of Groningen, University Medical Center Groningen, Department of Endocrinology, Groningen, The Netherlands; b Isala, Department of Clinical Chemistry, Zwolle, The Netherlands; c Isala, Department of Innovation and Science, Zwolle, The Netherlands; d University of Groningen, University Medical Center Groningen, Department of Internal Medicine, Groningen, The Netherlands; e Isala, Diabetes Research Center, Zwolle, The Netherlands

**Keywords:** CABG, Coronary Artery Bypass Grafting, CGM, Continuous Glucose Monitoring, CVA, Cerebral Vascular Event, DM, Diabetes Mellitus, DVN, Diabetes Vereniging Nederland, EQ-5D-3L, The 3-level version of EuroQol 5, FLARE-NL, FLAsh monitor Registry in The Netherlands, FSL-FGM, Free Style Libre Flash Glucose Monitor, HRQoL, Health Related Quality of Life, IQR, Interquartile Range, LADA, Latent Autoimmune Diabetes in Adults, MODY, Maturity-Onset Diabetes of the Young, OBGLD, Oral Blood Glucose Lowering Drugs, PCI, Percutaneous Coronary Intervention, Rt-CGM, Real time Continuous Glucose Monitoring, SD, Standard Deviation, SF-12^v2^, 12-Item Short Form Health Survey ^v2^, SMBG, Self-Monitoring of Blood Glucose, TIA, Transient Ischemic Attack, ZK, Zilveren Kruis (Insurance company), Type 1 diabetes, FreeStyle Libre, Flash glucose monitoring, Continuous glucose monitoring

## Abstract

**Aims:**

To identify factors predicting HbA1c reduction in patients with diabetes mellitus (DM) using FreeStyle Libre Flash Glucose Monitoring (FSL-FGM).

**Methods:**

Data from a 12-month prospective nation-wide FSL registry were used and analysed with multivariable regression. For the present study we included patients with hypoglycaemia unawareness or unexpected hypoglycaemias (n = 566) and persons who did not reach acceptable glycaemic control (HbA1c > 70 mmol/mol (8.5%)) (n = 294). People with other indications for use, such as sensation loss of the fingers or individuals already using FSL-FGM or rtCGM, were excluded (37%).

**Results:**

Eight hundred and sixty persons (55% male with a mean age of 46.7 (±16.4) years) were included. Baseline HbA1c was 65.1 (±14.5) mmol/mol (8.1 ± 1.3%), 75% of the patients had type 1 DM and 37% had microvascular complications. Data concerning HbA1c was present for 482 (56.0%) at 6 months and 423 (49.2%) persons at 12 months. A significant reduction in HbA1c (≥5 mmol/mol (0.5%)) was present in 187 (22%) persons. For these persons, median HbA1c reduction was −9.0 [−13.0, −4.0] mmol/mol (−0.82 [−1.19, −0.37]%) at 6 months and −9.0 [−15.0, −7.0] mmol/mol (−0.82 [−1.37, −0.64]%) at 12 months. In multivariable regression analysis with age, gender and SF-12 physical and mental component scores as covariates, only baseline HbA1c was significant: −0.319 (SE 0.025; p < 0.001; R^2^ = 0.240 for the model). In exploratory analysis among subgroups with different indications for FSL-FGM use (hypoglycaemia unawareness or persistently high HbA1c) and persons with a significant HbA1c decrease over the study period, baseline HbA1c remained the only significant predictor.

**Conclusions:**

Among the variables we analysed in the present study, only high HbA1c at baseline predicts significant HbA1c reduction during FSL-CGM use.

## Introduction

Accurate glucose monitoring is of utmost importance for persons with diabetes mellitus (DM) in order to achieve optimal metabolic control and thus avoid or delay the development of micro- and macrovascular complications, and maintain quality of life [1], [2]. HbA1c is considered to render a reasonably accurate representation of the degree of metabolic control: the lower the HbA1c, the better the average glucose control. However, low HbA1c levels are often accompanied with an increased occurrence of hypoglycaemic episodes. Finding a good balance between adjusting insulin doses, energy intake, and other lifestyle factors influencing blood glucose levels is therefore important.

Classically, self-measurement of blood glucose (SMBG) is based on fingerprick testing. However SMBG only provides information about a single timepoint, and often is painful and cumbersome. Therefore, during the last decades, realtime continuous glucose monitoring (rt-CGM) has been introduced. This system allows a semi-continuous insight, not only in glucose concentrations, but also in trends in time. Furthermore, when combined with continuous subcutaneous insulin infusion (CSII), it allows automated alarms and even adjustments of insulin doses according to the registered interstitial glucose concentrations. During the last years, Flash Glucose Monitoring (FGM) using the Free Style Libre (FSL, Abbott) system was introduced as an alternative for SMBG. The FSL-FGM consists of a sensor, via a needle inserted in the interstitial fluid, and as a patch placed on the back of the upper-arm. Upon scanning the sensor with a reader device it provides semi-continuous information about interstitial glucose concentrations. A recent study showed reasonable accuracy of FSL-FGM arm sensor readings demonstrated against capillary values [3].

Several studies demonstrated that the use of FSL-CGM results in better glycaemic control among persons with type 1 and type 2 DM. Tyndall *et al.* reported among 900 persons with type 1 DM a mean HbA1c reduction of 4 mmol/mol (0.37%) during a period of 245 days with FSL-FGM [4]. Nana *et al.* showed that initiation of FSL-FGM in their hospital (n = 90) resulted in a mean HbA1c decrease of 7 mmol/mol (0.64%) over a mean follow-up time of 4.6 months [5]. Recently, our research group reported the one-year results of the nation-wide prospective registry of FSL-FGM use in the Netherlands (FLAsh monitor Registry in The Netherlands, FLARE-NL). Besides a mean HbA1c reduction of 4 mmol/mol (0.37%) (even with less reported hypoglycaemic periods), there was also a reduction in work absenteeism rate, diabetes related hospital admissions, and a marked improvement in quality of life (QoL) [6].

It should be noted however, that the suitable target population most likely to benefit from the FSL-FGM with regards to HbA1c improvement is not yet known. Of course, it stands to reason to expect the largest improvement in users with the highest baseline HbA1c levels. Indeed, in the study by Tyndall *et al.* higher baseline HbA1c (≥58 mmol/mol (7.5%)) was a predictor of an HbA1c fall of ≥5 mmol/mol (0.5%), whilst older age at diagnosis was independently associated with non-response [4].

As such, the aim of the present study is therefore to provide more evidence to identify patients who are likely to benefit from the use of FSL-FGM with regard to their HbA1c levels. For this purpose we used data from the Flash monitor registry in the Netherlands (FLARE-NL), a nation-wide prospective registry of persons with DM using FSL-FGM.

## Patients and methods

The FLARE-NL registry has a prospective, observational design and aimed to assess the effects of use of the FSL-FGM on clinically relevant endpoints, with emphasis on HbA1c (primary outcome), but also changes in frequency and severity of hypoglycaemia, Health Related Quality of Life (HRQoL), and experienced disease burden over a period of 1 year [7]. The study protocol was registered at the Dutch trial register (www.trialregister.nl (NTR6212)). Outcomes for all participants are published previously. The aim of the present analysis was to investigate, in a post-hoc analysis, variables that predict HbA1c decline among persons with type 1 DM during use of FSL-FGM.

Adults (≥18 years) with DM using insulin were eligible for participation in the FLARE-NL registry. All subjects were treated by a hospital-based diabetes team, had a health insurance with the Dutch insurance company Zilveren Kruis (ZK) and belonged to one or more pre-specified targets groups. The definitions of these target groups (indications for FSL-FGM use) were formulated in cooperation with a patient panel and the Dutch diabetes patient organisation, the Diabetes Vereniging Nederland (DVN). These original indications were described in detail previously. For the present analyses we only included persons with hypoglycaemia unawareness (156, original indication number 1), unexpected hypoglycaemias despite an average of 6 or more measurements per day (410, original indication number 2) and persons who did not reach acceptable glycaemic control, as evidenced by a mean HbA1c > 70 mmol/mol (8.5%) over the year preceding the inclusion (294, original indication number 3). As such, from the available population of 1365 subjects, 19 (original indication number 4 i.e. individuals with sensation loss of the fingers), 57 (original indication number 5 i.e. individuals with occupational hazards), 45 (original indication number 6 i.e. persons already using rt-CGM), 100 (original indication number 7 i.e. individuals already using FSL) and 284 (individuals with multiple indications for FSL) subjects (in total 505) were excluded. Therefore, 860 subjects (63%) of the initial total study population were included in the present analyses.

Detailed information concerning the FLARE-NL registry has been published previously [7]. In brief, the departments of Internal Medicine and/or Diabetes Centers of all 95 hospitals in the Netherlands were invited to include individuals based on the inclusion criteria as described above. At baseline, informed consent of the intended FSL-FGM user was obtained. Next, the participant received a link to fill out the various questionnaires in the online registry. The healthcare provider filled out the data necessary for the registry. These data included demographics (age, gender), type of DM, indication for participation, level of HbA1c (preceding 4 values), presence of microvascular (neuropathy, nephropathy, retinopathy) or macrovascular complications, frequency of SMBG, number of DM-related hospitalizations, number of hypoglycaemic events, absenteeism rate and working day losses or reduced functioning due to DM. Furthermore, participants were asked to complete questionnaires related to HRQoL including the 12-Item Short Form Health Survey ^v2^ (SF-12; physical and component scores (PCS and MCS) were calculated) and the 3-level version of EuroQol 5D (EQ-5D-3L; with scores on a tariff scale and a visual analogue scale (VAS)) [8], [9], [10].

After 6 and 12 months participants and healthcare providers were asked to report HbA1c results from the preceding 6 months, In addition, participants were asked to report changes in presence of complications, the number of diabetes-related hospitalizations in the previous period, hypoglycaemias (<3 mmol/L) in three months before filling out questionnaires, work absenteeism rate in prior 6 months or reduced functioning (including sports performance) due to dysregulation of DM, and the HRQoL questionnaires.

Results are expressed as mean (with standard deviation (SD)) or median (with interquartile range [IQR]) for normally distributed and non-normally distributed data, respectively. Normality was examined with Q-Q plots. Variables with a skewed distribution were log_10_ transformed before analysis. We defined a clinically significant HbA1c decrease as a HbA1c difference of ≥5 mmol/mol (0.5%) between baseline and the last available HbA1c concentration, according to the NICE guideline, the analysis by Tyndall *et al.* and taking into account the documented variability in HbA1c measurements [11], [12].

Univariate analyses for correlation were performed to investigate the association between the difference in HbA1c over the study period and other variables. Variables with a *p* value < 0.1, not corrected for multiple testing, were checked for confounding by performing partial correlation analyses. Next, multivariable linear regression analysis (simultaneous entry method) was performed to investigate associations between the difference in HbA1c over the study period as dependent variable and multiple independent covariates. Age, gender, baseline HbA1c and baseline SF-12 MCS and PCS scores were included as covariates in the multivariate model with the difference in HbA1c as dependent variable, based on previous literature [13]. Furthermore, covariates were included in the multivariable model in case the *p* value was ≤0.1 in the univariate analysis. The models were checked for collinearity.

As exploratory analysis, uni- and multivariable analyses were repeated in subgroups: (I) persons who started FSL use because of frequent unexpected hypoglycaemia, or hypoglycaemia unawareness, (II) persons who started FSL use because of inability to reach acceptable glycaemic control and (III) among persons who, during the 1-year duration of the FSL registry study, reached a clinically relevant HbA1c reduction.

A two-sided *p* value < 0.05 was considered statistically significant. All statistical analyses were performed with SPSS software (IBM SPSS Statistics for Windows, Version 26.0. Armonk, NY: IBM Corp.).

## Results

Baseline characteristics of the 860 subjects included in the present analysis are presented in Table 1. In brief, 470 (54.7%) was male, mean age was 46.7 (±16.4) years, 643 (74.8%) persons had type 1 DM, 161 (18,7%) type 2 DM and 56 (6,5%) other forms of DM. Baseline HbA1c was 65.1 (±14.5) mmol/mol (8.1 ± 1.3%). Three hundred and sixteen (36.7%) patients had a history of microvascular complication(s) at baseline and 125 (14.5%) a history of macrovascular complication(s).

**Table 1:** Baseline characteristics of all persons (n = 860) included in the present analysis.

	**All persons**
Male gender, n (%)	470 (54.7)
Age	46.7 (16.4)
HbA1c (mmol/mol)	65.1 (14.5)
HbA1c (%)	8.1 (1.3)
Strips use per day, n	6.1 (3.1)
Presence of any hypoglycaemic events in past 6 months, yes, n (%)	799 (92.9)
Absenteeism rate in past 6 months, yes, n (%)	147 (17.1)
Hospital admissions in past 12 months, yes, n (%)	120 (14.0)
**Type of diabetes**	
Type 1 diabetes	643 (74.8)
Type 2 diabetes	161 (18.7)
LADA	39 (4.5)
MODY	4 (0.5)
Other forms of diabetes	13 (1.5)
**Therapy**	
Insulin monotherapy	702 (81.6)
Insulin and OBGLD	158 (18.4)
**Complications**	
Presence of microvascular complications, n (%)	316 (36.7)
Neuropathy, n (%)	163 (19.0)
Albuminuria, n (%)	168 (19.5)
Retinopathy, n (%)	173 (20.1)
Presence of macrovascular complications, n (%)	125 (14.5)
Angina pectoris, n (%)	23 (2.7)
PCI, n (%)	33 (3.8)
Myocardial infarction, n (%)	21 (2.4)
CABG, n (%)	24 (2.8)
TIA, n (%)	16 (1.9)
CVA, n (%)	12 (1.4)
Peripheral arterial disease, n (%)	35 (4.1)
**QoL**	
SF-12 PCS	50.5 [44.6, 54.1]
SF-12 MCS	48.9 [40.3, 56.4]
EQ5D Dutch tariff	0.84 [0.77, 1.00]
EQ5D VAS	71.0 [61.0, 81.0]

Data in the second column are presented as number (%), mean (SD) or median [25, 75th percentile]. Abbreviations: CABG, coronary artery bypass grafting; CVA, cerebral vascular event; MCS, mental component scale; OBGLD, oral blood glucose lowering drugs; PCI, percutaneous coronary intervention; PCS, physical component scale, TIA, transient ischemic attack.

In the total population, data concerning HbA1c was present for 482 (56.0%) at 6 months and 423 (49.2%) at 12 months. For these patients, the median change in HbA1c was −3.0 [−9.0, 1.0] (−0.27 [−0.82, 0.09]%) and −3.0 [−8.0, 2.0] mmol/mol (−0.27 [−0.73, 0.18]%) at 6 and 12 months respectively. A significant reduction of HbA1c (of ≥5 mmol/mol (0.5%)) was present in 187 (22%) persons. For these persons the median HbA1c reduction was −9.0 [−13.0, −4.0] mmol/mol (−0.82 [−1.19, −0.37]%) at 6 months and −9.0 [−15.0, −7.0] mmol/mol (−0.82 [−1.37, −0.64]%) at 12 months.

**Fig. 1:**
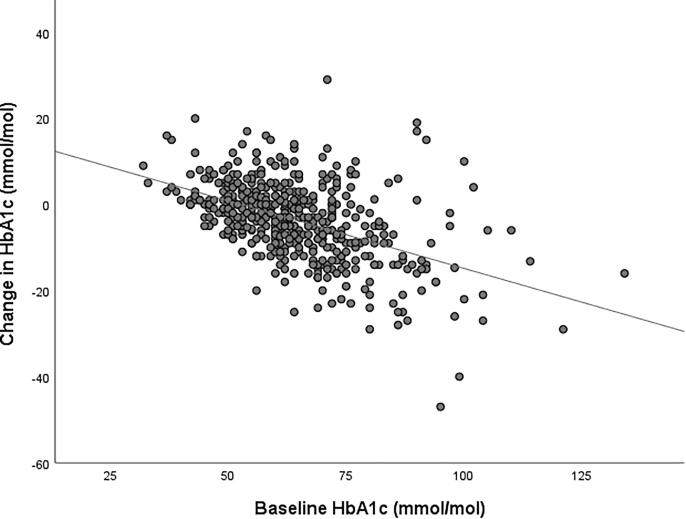
Relationship between baseline HbA1c and delta HbA1c. Legend: the relationship between baseline HbA1c concentrations and the 12-month change in HbA1c following start of FSL-FGM (n = 423). Pearson r −0,490, p < 0.001.

Besides baseline HbA1c (r = −0.490, p < 0.001) (Fig. 1) none of the variables in Table 1 was significantly associated with delta HbA1c over the study period in univariate analysis (data not shown). In multivariable analysis (See Table 2) with age, gender, SF-12 PCS and MCS scores as other covariates, only baseline HbA1c proved to be the significant predictor: −0.319 (SE 0.025, p < 0.001; R^2^ = 0.240 for the model).

**Table 2:** Multivariable analysis for delta HbA1c.

	Unstandardized B (SE)	p-value
Age (years)	−0.023 (0.024)	0.331
Gender (1 = male)	0.121 (0.708)	0.917
Baseline HbA1c mmol/mol	**−0.319 (0.025)**	<0.001
SF-12 PCS	−0,028 (0.049)	0.245
SF-12 MCS	0.030 (0.034)	0.384

Multivariable linear regression model. Explained variance R^2^ = 0.240. Significant outcome presented in bold.

In exploratory multivariable analysis amongst the subgroups of persons who started FSL-FGM because of hypoglycaemia unawareness (group I), persistently high HbA1c (group II) and persons who had reached a significant HbA1c reduction over the study period (group III), baseline HbA1c remained the only predictor of the difference in HbA1c over the study period (See Supplement).

## Discussion

In this study we aimed to identify factors that are associated with improvement of HbA1c among persons with DM using FSL-FGM. In both the total population and in different subgroups (i.e. patients with hypoglycaemia unawareness, persistently high HbA1c or significant HbA1c reduction over study period) baseline HbA1c was the single factor predictive of HbA1c decline.

In our previous study, that reported changes in HbA1c when using FSL-FGM and included a larger (though more unselected) subset of patients included in the Dutch FSL-FGM registry, the greatest HbA1c decline was measured in the group with inadequate glycaemic control (HbA1c > 70 mmol/mol (8.5%)) (6). The current study emphasizes this and does not identify other predictors of HbA1c decline. Tyndall *et al.* presented a comparable strong negative correlation between baseline HbA1c and subsequent change in HbA1c (r −0.479) with FSL-FGM use among 900 patients with type 1 DM [13]. Similar to Tyndall *et al.* we found no association between age or sex and change in HbA1c. Furthermore, we found no relation between change in HbA1c and type of DM, number of strips used per day (SMBG) prior to start of FSL-FGM, presence or absence of micro- or macrovascular complications, and quality of life.

Interestingly, we did not observe an association between the frequency of self-monitoring of blood glucose prior to FSL-FGM use and the decrease in HbA1c. Nevertheless, the amount of SMBG with fingerpricks is often used as a criterion for reimbursement of FSL-FGM (also in the Netherlands [14]). In the study by Tyndall *et al.* persons who performed SMBG prior to FSL-FGM use fewer than four times per day more often had a significant fall in HbA1c as compared to persons who did not: 67.7% vs. 45.3% (p < 0.01). Dunn *et al*. showed a clear association between frequency of FSL-FGM glucose scans and improvement in glycaemic parameters (consistent across different countries) [15]. Although hypothetical, this may implicate that (I) for a proportion of patients the use of FSL-FGM stimulates self-control (and thereby improvement of glycaemic control can be achieved) and (II) therefore the amount of SMBG with finger pricks prior to FSL-FGM use is not a valid criterion for FSL-FGM reimbursement.

Obviously, differences in study populations and health-care settings should be taken into consideration when comparing our results with other studies. In the study population of Tyndall *et al*. FSL-FGM was funded by the NHS (since 2017) for all persons who were using intensive insulin therapy and agreed to scan glucose levels at least six times a day. In the present study, however, patients had to finance half of the cost of the FSL-FGM themselves, because at that time the FSL-FGM was not reimbursed by the Dutch healthcare authorities and insurance companies. This resulted in a high drop-out rate; financial constraints were the most reported reason (55%). Although speculative, differences in reimbursement criteria may have resulted in a more determined population and thus more pronounced HbA1c reductions. Recently, the Dutch Institute of Care (ZorgInstituut Nederland, ZIN) published their decision on FSL-FGM, allowing use by the vast majority of people with type 1 DM and a selected group of people with type 2 DM [14]. It will be important to assess the eventual effects of this sweeping decision of use on eventual outcomes, amongst others HbA1c levels.

Other limitations of this study should be mentioned. First and foremost, this study lacks a control group. Many data were missing in this real life database. Since participation in the registry was voluntary, efforts to gain (more) information only partly succeeded. In addition, the present population is not extensively characterized. For instance, our dataset lacks data concerning age at diagnosis. As older age at diagnosis was associated with HbA1c non-response in the study by Tyndall *et al*. non-measured variables cq. confounding should be taken into consideration when interpreting this study. Furthermore, the use of strips per day prior to start of FSL-FGM was used as a proxy of frequency of SMBG in the current study. Although this difference may be a little bit semantic here, it could have resulted in an overestimation of the frequency of SMBG. As data were patient-reported, recall bias may be present. Importantly, the current population was a selection of the original FLARE-NL database, which may implicate selection bias. Finally, as participants had to finance half of the costs of the FSL-FGM themselves; this inevitably will contribute to selection bias, since the actual participants probably will be more affluent than the average DM population, at least in the Netherlands.

## Conclusions

In summary, a high baseline HbA1c is associated with a more pronounced HbA1c decrease with FSL-FGM use. No other predictive factors of clinically important reduction in HbA1c levels could be identified in this study; both in the total study population and in different subgroups.

## Funding statement

This study was supported by an unconditional grant from the Stichting Achmea Gezondheidszorg (SAG). The SAG is an innovation fund of health insurance company Zilverenkruis. The manufacturer of the Free Style Libre (Abbott) did not provide any finances for the proposed study and did not have any influence on study design, nor on definition of the target groups or the study objectives.

## Author contributions

AL: Statistical analysis, interpretation of data, writing manuscript. MJF: Collecting data, critically reviewing manuscript. MAE: Interpretation of data, critically reviewing manuscript. RJS: Critically reviewing manuscript. HJGB: Design, critically reviewing manuscript. PRvD: Design, statistical analysis, interpretation of data, critically reviewing manuscript. All authors approved the final version of the manuscript.

## Declaration of Competing Interest

The authors declare that they have no known competing financial interests or personal relationships that could have appeared to influence the work reported in this paper.
