# Supplementary material for: Determinants of HbA1c reduction with FreeStyle Libre flash glucose monitoring (FLARE-NL 5)
Source: J Clin Transl Endocrinol. 2020 Oct 12;22:100237. doi: 10.1016/j.jcte.2020.100237 (PMC7578738; doi:10.1016/j.jcte.2020.100237)
Supplement: Supplementary data 1 [file mmc1.docx]

**Supplement**

**A.** Univariate analyses amongst different subgroups.

|  | **Subgroup I**  (n=566) | **Subgroup II**  (n=294) | **Subgroup III**  (n=187) |
| --- | --- | --- | --- |
| Gender (male = 1) | -0.024 | 0.040 | 0.093 |
| Age (years) | -0.035 | 0.016 | 0.096 |
| HbA1c (mmol/mol) | **-0.474 **** | **-0.313 **** | **-0.552 **** |
| Strips use per day (n) | **0.103** | -0.090 | 0.030 |
| Presence of any hypoglycaemic events in past 6 months (yes) | -0.056 | -0.011 | **0.247 **** |
| Work absenteeism in past 6 months (yes) | -0.055 | 0.032 | **-0.185 *** |
| Hospital admissions in past 12 months (yes) | -0.056 | -0.002 | - 0.108 |
| **Type of diabetes** |  |  |  |
| Type 1 diabetes | 0.038 | -0.011 | **0.137** |
| Type 2 diabetes | -0.038 | -0.005 | **-0.205 **** |
| LADA | -0.007 | 0.023 | 0.120 |
| MODY | -0.087 | **0.162 *** | -0.025 |
| Other forms of diabetes | 0.033 | -0.112 | -0.113 |
| **Therapy** |  |  |  |
| Insulin monotherapy (yes) | -0.058 | -0.142 | 0.031 |
| **Complications** |  |  |  |
| Presence of microvascular complications (yes) | **-0.097** | **0.028** | **-0.149 *** |
| Presence of macrovascular complications (yes) | -0.011 | -0.065 | 0.004 |
| **QoL** |  |  |  |
| SF-12 PCS | 0.027 | 0.021 | 0.079 |
| SF-12 MCS | 0.032 | 0.036 | 0.051 |
| EQ5D Dutch tariff | 0.010 | -0.095 | 0.061 |
| EQ5D VAS | 0.064 | -0.074 | 0.107 |

* p<0.05 ** p<0.01. Bold: p<0.1. NA: not applicable. Subgroups: (I) persons who started FSL use because of frequent unexpected hypoglycaemia or hypoglycaemia unawareness, (II) persons who started FSL use because of inability to reach acceptable glycaemic control and (III) persons who reached a significant HbA1c reduction during the 1-year duration of the FSL registry study.

**B.** Multivariable regression analysis for HbA1c among subgroups

**Subgroup 1:** Persons who started FSL use because of frequent unexpected hypoglycaemia or hypoglycaemia unawareness.

|  | Unstandardized B (SE) | p-value |
| --- | --- | --- |
| Age (years) | -0.001 (0.025) | 0.971 |
| Male gender (1=male) | -0.177 (0.743) | 0.812 |
| HbA1c mmol/mol | -0.379 (0.039) | <0.001 |
| Strips use per day | 0.097 (0.146) | 0.504 |
| Presence of microvascular complications (yes) | -0.329 (0.845) | 0.697 |
| SF-12 MCS | 0.024 (0.036) | 0.503 |
| SF-12 PCS | -0.004 (0.054) | 0.943 |

Explained variance R^2^= 0.236

**Subgroup 2:** Persons who started FSL use because of inability to reach acceptable glycaemic control.

|  | Unstandardized B (SE) | p-value |
| --- | --- | --- |
| Age (years) | -0.030 (-0.059) | 0.616 |
| Male gender (1=male) | 0.289 (1.642) | 0.861 |
| HbA1c mmol/mol | -0.274 (0.070) | <0.001 |
| MODY | 17.6 (10.1) | 0.084 |
| Presence of microvascular complications (yes) | 0.663 (1.735) | 0.703 |
| SF-12 MCS | 0.017 (0.082) | 0.837 |
| SF-12 PCS | -0.033 (0.115) | 0.777 |

Explained variance R^2^= 0.123

**Subgroup 3:** Persons with HbA1c reduction over study period.

|  | Unstandardized B (SE) | p-value |
| --- | --- | --- |
| Age (years) | 0.030 (0.025) | 0.220 |
| Male gender (1=male) | 0.868 (0.663) | 0.192 |
| HbA1c mmol/mol | -0.256 (0.025) | <0.001 |
| Presence of any hypoglycaemic events in past 6 months (yes) | 3.329 (1.781) | 0.064 |
| Work absenteeism | -1.808 (0.912) | 0.048 |
| Presence of microvascular complications (yes) | -0.839 (0.742) | 0.260 |
| Type 1 diabetes | 1.817 (1.449) | 0.211 |
| Type 2 diabetes | -0.635 (1.645) | 0.700 |
| SF-12 MCS | 0.019 (0.034) | 0.575 |
| SF-12 PCS | -0.048 (0.046) | 0.301 |

Explained variance R^2^= 0.377

**Bovenkant formulier**
